# Large Nanodiscs: A Potential Game Changer in Structural Biology of Membrane Protein Complexes and Virus Entry

**DOI:** 10.3389/fbioe.2020.00539

**Published:** 2020-06-12

**Authors:** Krishna M. Padmanabha Das, William M. Shih, Gerhard Wagner, Mahmoud L. Nasr

**Affiliations:** ^1^Department of Biological Chemistry and Molecular Pharmacology, Harvard Medical School, Boston, MA, United States; ^2^Wyss Institute for Biologically Inspired Engineering at Harvard, Boston, MA, United States; ^3^Department of Cancer Biology, Dana-Farber Cancer Institute, Boston, MA, United States; ^4^Renal Division and Engineering in Medicine Division, Department of Medicine, Brigham and Women's Hospital, Harvard Medical School, Boston, MA, United States

**Keywords:** nanodisc, membrane protein, viral entry, membrane mimetic, DNA-corralled nanodisc, phospholipid bilayer, lipid-protein interactions, membrane protein complex

## Abstract

Phospho-lipid bilayer nanodiscs have gathered much scientific interest as a stable and tunable membrane mimetic for the study of membrane proteins. Until recently the size of the nanodiscs that could be produced was limited to ~ 16 nm. Recent advances in nanodisc engineering such as covalently circularized nanodiscs (cND) and DNA corralled nanodiscs (DCND) have opened up the possibility of engineering nanodiscs of size up to 90 nm. This enables widening the application of nanodiscs from single membrane proteins to investigating large protein complexes and biological processes such as virus-membrane fusion and synaptic vesicle fusion. Another aspect of exploiting the large available surface area of these novel nanodiscs could be to engineer more realistic membrane mimetic systems with features such as membrane asymmetry and curvature. In this review, we discuss the recent technical developments in nanodisc technology leading to construction of large nanodiscs and examine some of the implicit applications.

## Introduction

Membrane proteins (MPs) play critical roles in health, disease and hence in drug design. A majority of approved systemic drugs target membrane proteins due to their involvement in a variety of cellular processes such as signal transduction, transport of ions and molecules across the cell membrane, and cell adhesion to surfaces (Cournia et al., 2015). Structural information on MPs is a key benefit when seeking new therapeutics, and major efforts have been made toward this aim in the recent years (Bill et al., 2011; Cheng, 2018). However, MPs represent a very small fraction of the available protein structures in the Protein Data Bank (PDB), mainly due to challenges in preparing functionally relevant samples in sufficient quantities. So far, the most popular membrane mimetics used in membrane protein structural biology are detergent micelles (Linke, 2009). Even though detergents are easy to handle, they are not ideal for membrane proteins as they may affect sensitive tertiary structure, reduce the MPs' stability and may cause unfolding of the proteins. Detergents can also interfere with the binding of interaction partners as well as with subunit assembly in membrane protein complexes. Phospholipid liposomes and supported bilayers are other popular membrane mimetics that can harbor membrane proteins in a detergent-free lipid environment, however, they tend to be very non-homogeneous and are not compatible with most of the high resolution structural biology techniques.

Phospholipid nanodiscs are known to provide a native-like membrane environment that aids in preparing integral membrane proteins in biologically active folded forms for structural studies (Denisov et al., 2004; Ritchie et al., 2009). The majority of the nanodiscs are constructed exploiting the unique property of the α-helical-amphipathic proteins derived from apolipoprotein A-I that wrap around the lipid bilayer as a homo dimer. These amphipathic proteins are commonly called membrane scaffold proteins (MSP). There are other nanoscale phospholipid bilayer systems such as steyrene-maleic acid (SMA) copolymer lipid discs (Knowles et al., 2009) and a saposine-A lipoprotein nanoparticle system (Frauenfeld et al., 2016); however, a detailed discussion about these approaches is beyond the scope of this review.

**Figure 1:**
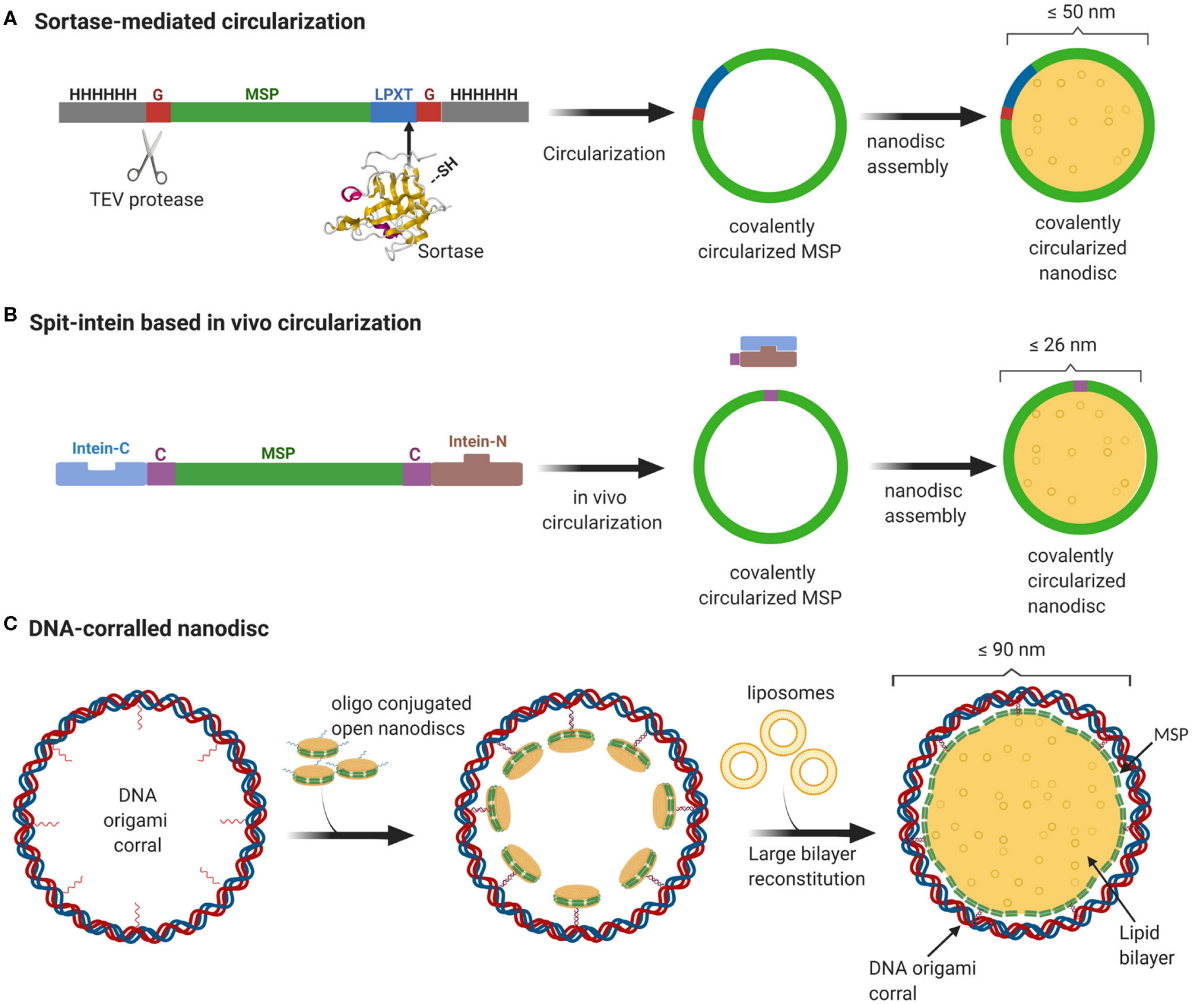
Overview of currently available methods to produce stable and homogeneous large nanodiscs. **(A)** Sortase based MSP circularization strategy. Sortase cleaves between the Gly and Thr of the LPXTG motif at the C-teminus of MSP and catalyzes the formation of an amide bond with the N-terminal Gly resulting in a circular MSP which can be used to assemble nanodiscs of sizes ranging from 9 to 50 nm. **(B)** Split intein-based strategy for circularization of MSP *in vivo* using Npu DnaE split-intein. MSP fusion results in nanodiscs as big as 26 nm. **(C)** Outline of DNA-corralled nanodisc protocol. Small nanodiscs functionalized with oligos bind to specified sites on the DNA origami resulting in a small nanodisc-decorated barrel. Addition of detergents and lipids, followed by removal of detergents using dialysis, leads to reconstitution of large nanodiscs within DNA barrel.

Even though MSP-based nanodiscs are widely used, they faced several challenges including heterogeneity of the sample resulting from both the nanodisc size as well as the number of copies of membrane proteins enclosed. As one would expect, the extent of heterogeneity is even more pronounced for nanodiscs with larger diameter. Recently, we developed a method to stabilize the scaffold protein and to overcome the size heterogeneity by covalently linking the termini of the scaffold proteins. This sortase mediated reaction enabled us to make covalently circularized nanodiscs (cNDs) of size up to 50 nm without compromising on the sample homogeneity (Nasr et al., 2017) (Figure 1A). Further efforts to make larger stable cNDs turned out to be futile, mainly because of their tendency to aggregate. Furthermore, it was challenging to produce large scaffold proteins in *E.coli* expression systems mainly due to very low yield.

In a more recent effort, we used a DNA origami scaffold to support and stabilize nanodiscs of up to 70 nm inner diameter (Zhao et al., 2018). We employed DNA origami barrels as scaffolding corrals to recruit small non-circularized nanodiscs to the inner wall, so that upon addition of excess lipid, the neighboring small nanodiscs merge together to form one single large bilayer surrounded by MSP helices and an outside DNA corral (Figure 1C). In this review, we strive to give a brief sketch of the design and recent applications of these large nanodiscs, and their potential for vast applications ranging from studying membrane-viral interactions to controlled reconstitution of large multi-protein complexes in bilayers.

## Design and Engineering of Large Nanodiscs

In a typical nanodisc, two copies of MSP wrap around the lipid bilayer where the diameter of the nanodisc is mainly determined by the length of the MSP. Our research group had previously developed recombinant versions of MSP that can be circularized using sortase and enable the assembly of stable and homogeneous nanodiscs of varying sizes from 8.5, 11, 15, and up to 50 nm namely NW9, NW11, NW30, and NW50, respectively (Nasr et al., 2017).

In a more recent work, we utilized DNA origami to enable the assembly of even larger nanodiscs. DNA origami is a method of building custom three-dimensional shapes using the M13 DNA in nanometer scale (Douglas et al., 2009). Here, large nanodiscs are produced by first decorating the DNA barrels with small lipid-bilayer nanodiscs, which open up to form large nanodiscs upon addition of excess lipids.

The enclosed nanodiscs are relatively stable and tolerant to a broad range of pH levels and divalent ion concentrations. This nanodisc system was used to reconstitute two membrane-protein clusters and to study poliovirus entry (Zhao et al., 2018).

## Recent Optimizations and Modifications

Recently another modification to the sortase based MSP circularization method was shown to be effective in avoiding high molecular weight byproducts and enhancing the yield (Yusuf et al., 2018). This protocol involves performing the sortase mediated ligation at room temperature and using detergents during the circularization. These circular nanodiscs were found to be comparable with the previously described circularized nanodiscs in terms of stability against heat denaturation and concentration-dependent aggregation.

A recent study demonstrates an easy *in vivo* approach for the MSP circularization in which the circularized proteins can be directly purified from the cells. This protocol has been shown to yield circularized nanodiscs of varying sizes from 8 to 26 nm with comparable size homogeneity and thermal stability to the sortase-based approach (Miehling et al., 2018) (Figure 1B). Another recent study aimed at continued development of the circularized nanodisc protocol developed an efficient method for large scale production of circularized MSP yielding 75 mg per liter of bacterial culture (Johansen et al., 2019). The main reason for this notable increase in yield was the sequence optimization of the original MSP sequence to incorporate extra negatively charged residues. This enhanced solubility dismisses the need or use of detergents in any of the purification or circularization steps.

## Experimental Considerations for Making Large Nanodiscs

In order to obtain high quality monomeric circularized products, we usually perform the circularization reaction at low protein concentrations. We keep the final concentrations of NW9 and NW11 at or below 20 μM. In case of NW30 and NW50, we perform the circularization at or below 2 μM.

The optimal ratio between the scaffold protein and lipid must be determined by a series of test assembly reactions and screening for sample homogeneity (Hagn et al., 2018). A quick method to approximate the right ratio is to calculate the area from the expected diameter and use a ratio of 3.2 molecules of lipid per nm^2^: For example, the NW50 construct which is 910 aa long will result in a nanodisc of diameter ~43.3 nm, and an area of ~1,450 nm2. This means one would need to use a NW50: lipid ratio of 1: 4640 to assemble empty nanodiscs. The surface area of a membrane protein can be predicted as well. For example, a single transmembrane (TM) helix has a diameter ranging from 0.6 to 1 nm, taking up around 8 nm^2^. This means a seven TM-helical protein, like a GPCR, would require around 56 nm^2^. These quick calculations could save time during the screening for the optimal ratio between proteins and lipids.

The DCND protocol involves folding of DNA origami, and then hybridizing oligo-coupled non-circularized nanodiscs to the DNA barrels followed by addition of excess lipids and dialysis, resulting in large DNA-barrel-scaffolded lipid nanodisc. We use a lipid mixture containing 10% cholesterol which is known to intercalate between the phospholipids and prevent the bilayer from clustering or stiffening. The membrane protein to be incorporated into DCND can be added early during the small oligo-coupled nanodisc assembly or can be added at a later step along with the excess lipids during the reconstitution of the large lipid bilayer. Using the latter approach, we were able to incorporate human Voltage-Dependent Anion channel 1 (hVDAC-1) and the *Rhodobacter sphaeroides* photosynthetic reaction center protein (RC) into DCND and present these proteins at a very high density and forming multimeric assemblies. This protocol could be further developed to form 2D crystals of membrane proteins which may be useful for membrane protein structure determination using techniques such as X-ray free electron laser or cryo-electron tomography (Schur et al., 2016).

## Structural Studies of Large Membrane Protein Complexes

There have been several structural studies of membrane proteins assembled in nanodiscs using Cryo-electron microscopy (Cryo-EM) which shed light into several critical biological processes. Transient receptor potential cation channel subfamily V (TRPV-1) is shown to be interacting with the lipids in the nanodisc causing the regulation of its ligand binding (Gao et al., 2016). The structures of the intact *Thermus thermophilus* V/A-ATPase, in multiple conformations in nanodisc studied by Cryo-EM indicated flexibility between V_1_ and V_*o*_ in a working enzyme, resulting in competition between central shaft rotation and resistance from the peripheral stalks (Zhou and Sazanov, 2019). Other notable Cryo-EM structures of membrane protein embedded in nanodiscs include the pore forming TcdA1 toxin subunit from *Photorhabdus luminescence* (Gatsogiannis et al., 2016), yeast oilgosaccharyltranferase (Wild et al., 2018). Some of the latest MP structures in nanodiscs include structures of the homohexameric Leucine Rich Repeat Containing 8 VRAC Subunit A (LRRC8A) channel in the presence and absence of the inhibitor DCPIB (Kern et al., 2019), the structure of the lipid scramblase transmembrane protein 16F (TMEM16F) which displays the coexistence of an intact channel pore and PIP2-dependent protein conformation changes leading to membrane distortion (Feng et al., 2019). One of the remarkable Cryo-EM structure is the structure of a ribosome secreting a newly synthesized protein chain through the protein export channel SecYE incorporated into nanodisc (Frauenfeld et al., 2011).

Even though structure determination of single membrane proteins using nanodisc technology has become more common, fewer structures of membrane protein complexes have been determined. The introduction of large cNDs and DCND has made it possible to study large membrane protein complexes using Cryo-EM as the size of the nanodisc can be critical to maintain a native oligomeric state. Earlier work by our group devised a method to co-reconstitution of multiple membrane proteins in a single nanodisc by temporarily attaching complementary oligo nucleotides to individual proteins (Raschle et al., 2015). This method enables the formation and insertion of detergent solubilized small membrane protein complexes into the nanodiscs with well-defined stoichiometry and precise control over individual proteins. Using this method highly pure nanodiscs containing either dimeric or trimeric protein complex of voltage-gated anion channel (VDAC) were produced and imaged by negative-stain EM. This method is ready to be utilized in the large cNDs and DCND assembly protocol. In the original origami design for 90 nm DCNDs there are 18 oligos which are meant to be connected to the oligo conjugated protein of interest or functionalized nanodisc (Zhao et al., 2018). These oligos can be custom designed according to the stoichiometry required for a particular membrane protein complex, and complex formation can be triggered by addition of excess lipids and subsequent reconstitution of large nanodisc.

## Virus Entry and Virus-Membrane Interactions

**Figure 2:**
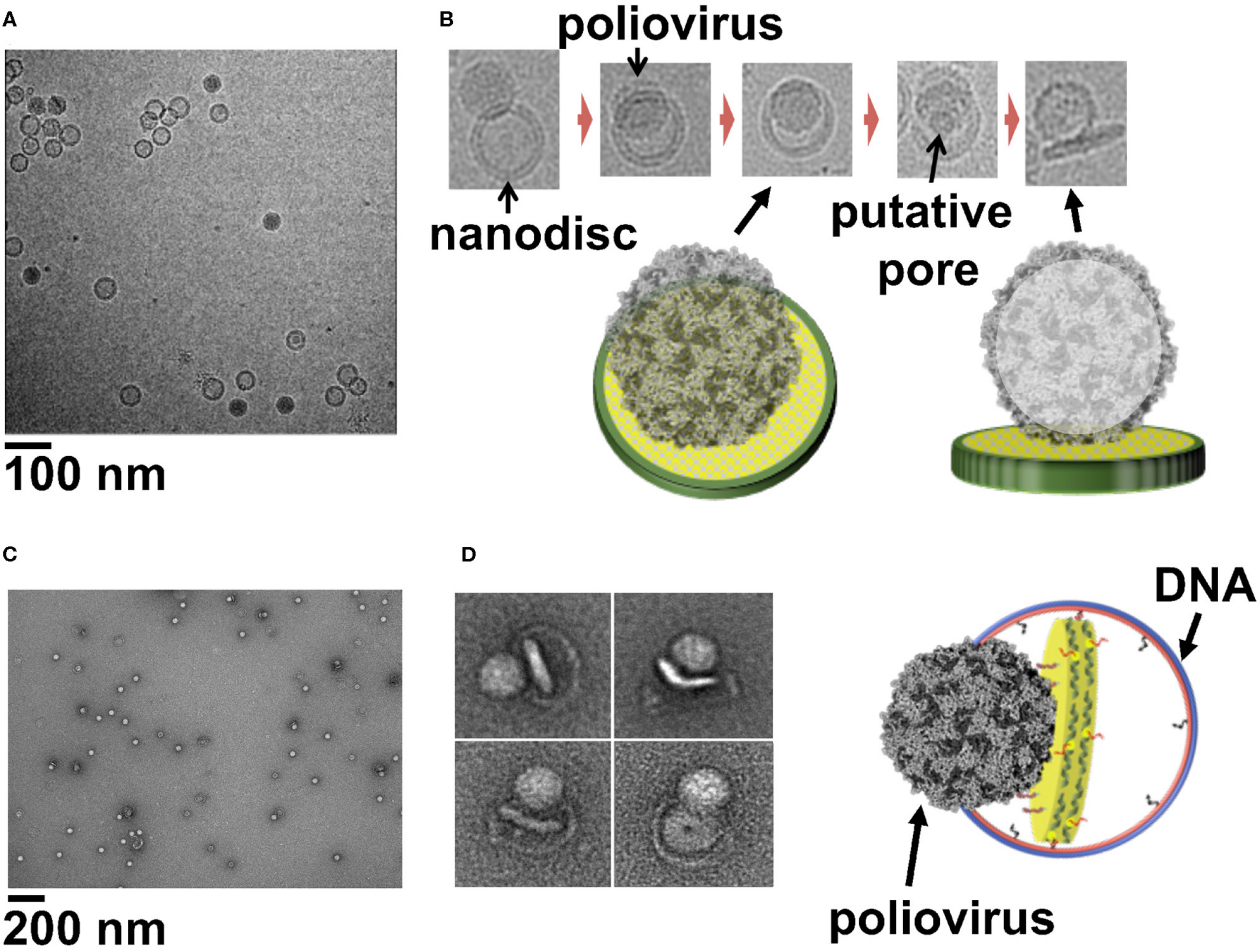
**(A)** Cryo-EM image of a field view of poliovirus interacting with 50 nm cND containing CD155 receptor. **(B)** Cryo-EM images of individual viral particle tethered to nanodisc containing CD155. **(C)** Negative-stain TEM images of poliovirus interacting with 60 nm DCND containing CD155 receptor. **(D)** TEM images of individual viral particles interacting with DCND containing CD155. Some of the nanodiscs were partially released from their DNA scaffolds after binding the virus. The images show bending of the bilayer and creation of a pore in nanodisc by the poliovirus. A Cartoon representation of a nanodisc partially released from its DNA scaffold upon binding to the virus (right). This figure is adapted and used with permission from references (Nasr et al., 2017; Zhao et al., 2018).

The entry of viruses inside the host cells require the disruption of the host cell membrane without disturbing the integrity of the cell. This crucial step of infection is orchestrated by embedded glycoproteins in envelope viruses (White and Whittaker, 2016), whereas in non-enveloped viruses this is carried out by hydrophobic capsid peptides (Banerjee and Johnson, 2008). Although extensive research has derived insights into viral membrane interaction and cellular entry, many questions remain unanswered regarding the mechanism of membrane penetration, disassembly of virus, conformational changes, and the role played by the host factors. However, visualizing the virus at the moment of membrane interaction is very challenging and remains the major hindrance in studying viral cell entry. Previous cryo-electron tomography studies using a receptor-decorated liposome system have demonstrated that poliovirus 135 s particles form 50 Å connectors, attaching the virus to the membrane and providing a passage to the viral genome and initiating the infection (Strauss et al., 2013). In case of rotavirus particles, Cryo EM-tomography showed direct interaction between VP4 spikes and the membrane (Abdelhakim et al., 2014). A similar study using liposome as a membrane model and human rhinovirus 2 (HRV2) particles, showed that the virus binds to the membrane around a 2-fold icosahedral symmetry axis (Kumar and Blaas, 2013). Even though these studies have elucidated the capsid transition and viral-membrane interaction, further studies are limited by the disadvantages of liposomes as a membrane model. Large nanodiscs with their available surface area, homogeneity and stability provide an attractive alternative to be used as surrogate membranes to study the step wise alterations leading to membrane entry by most viruses. In our previous work we used a 50-nm circularized nanodisc to study how poliovirus attaches to the membrane and transfer its genome into the host cell. EM images we obtained showed how poliovirus bound to 50 nm cNDs decorated with CD155 receptors, form a putative pore and ejects RNA across membrane (Figures 2A,B) (Nasr et al., 2017). Similarly, using 45-nm DCNDs functionalized with His-tagged CD155 ectodomain we were successful in initiating the receptor mediated uncoating of poliovirus and capturing glimpses of early steps of virus attachment to the bilayer (Figures 2C,D) (Zhao et al., 2018). The advantages of working with DCNDs in order to study viral-membrane interactions can be remarkable because of the unprecedented possibilities of functionalization with host cell receptors or receptor complexes.

## Other Potential Opportunities Offered by Large Nanodiscs

Vesicular transport pathways such as synaptic pathways are of great scientific interest. Synaptic vesicle carrying neurotransmitters are delivered by regulated fusion carried out by the action of Soluble NSF Attachment Protein Receptors (SNARE), vesicles (v)-SNARE (VAMP2/Synaptobrevin) folds with a plasma membrane-situated target (t)-SNARE to form SNARE pin which is a four-helical bundle (Sollner et al., 1993; Poirier et al., 1998). One of the examples of how large nanodiscs can aid in addressing previously inaccessible biological processes, is the recently published study of mechanisms that determine the size and kinetic properties of the fusion pore (Bao et al., 2018). In this particular study, 50 nm circularized nanodiscs were used to trap fusion pores in their initial open state, and with the newly devised ND-BLM assay they could directly prove that a certain number of Soluble NSF Attachment Protein Receptors (SNAREs) are needed to hold fusion pores open, and that more SNAREs result in larger pores. Since smaller nanodiscs could not have covered the larger surface area demand by the system, and the liposomes being non-homogeneous in size, this work would not have been achieved easily if not for large nanodiscs. It will be also interesting to study other fusion systems such as mitochondrial and ectoplasmic fusion using the large nanodiscs.

Supported lipid bilayers coupled to an elastic substrate were used to study the effects of the membrane adhesion on the area regulation of cell membrane. By tuning the strain of the substrate, the dynamical response of the strongly adhered bilayer to biaxial expansion and compression was observed (Staykova and Stone, 2011). This study using supported bilayer demonstrated that a lipid membrane subject to lateral stretching expands its area by incorporating vesicles. The vesicles act as a reservoir of lipids and the extent to which the membrane can expand was shown to be proportional to the number of vesicles attached. Even though this study reaffirms the observations on live cells, where an increased membrane tension causes membrane fusion (Hamill and Martinac, 2001), the large nanodiscs could provide a unique opportunity to study the membrane area regulation and the effect of membrane asymmetry on the cell surface area.

## Membrane Asymmetry and Curvature in Large Nanodiscs

Most of the natural cell membranes are known to exhibit membrane asymmetry in terms of lipid composition of the inner and outer leaflets. However, the majority of ongoing research utilizes chemically well-defined symmetric planner bilayers or liposomes for structural studies of membrane proteins. Eukaryotic plasma membrane actively segregates sphingomyelin (SM) and phosphatidylcholine (PC) in the outer membrane monolayer and phosphatidyl ethanolamine (PE), phosphatidylserine (PS), and phosphatidylinositol (PI) on inner monolayer (Verkleij, 1973; Devaux, 1991). Symmetric model membranes lack some of the key features of cell membranes which could be crucial for membrane interactions of the integral membrane proteins. An asymmetric bilayer model system will be beneficial to provide integral membrane proteins an environment very close to the native biological membranes. A recent protocol describes the preparation of unilamellar asymmetric phospholipid vesicles, using methyl -beta cyclodextrin which actively exchanges the outer leaflet (Doktorova et al., 2018). A similar method can be devised exploiting the tunability of DCND to make asymmetric nanodiscs either using methyl -beta cyclodextrin for creating a lipid asymmetry between the leaflets or by strategically placing flippases on the DCND to facilitate both the creation and maintenance of an asymmetric bilayer.

## Conclusions

This review provides a brief insight of how introduction of cNDs and DCNDs has enabled the possibility to construct very large nanodiscs, which has extended the application of nanodisc technology from structural biology of small membrane proteins to study of large membrane protein complexes and membrane interaction of viruses. We highlighted some of the still unexploited potentials of these stable large tunable bilayers, which could be applied to several branches of cellular structural biology. Large nanodiscs offer a versatile tool to study membrane interaction of viruses which can solve several questions about virus entry such as mechanism of membrane penetration, steps in disassembly of virus particles, conformational changes, and the regulatory role played by host cell surface receptors. Large nanodiscs can also aid in understanding several biological processes such as membrane and vesicular fusion, and can be further developed to engineer asymmetric nanodiscs which will provide a more realistic membrane mimetic.

## Author Contributions

KP, WS, GW, and MN wrote the manuscript. MN and KP drew the figures. All authors approved the final manuscript.

## Conflict of Interest

GW and MN have founded NOW Scientific to allow purchase of covalently circularized scaffold proteins. Plasmids are available for a nominal fee, and detailed protocols have been published. Figure 1 is created using BioRender. The remaining authors declare that the research was conducted in the absence of any commercial or financial relationships that could be construed as a potential conflict of interest.

## Abbreviations

cND: covalently circularized nanodiscs
DCND: DNA corralled nanodiscs
GPCR: G protein-coupled receptor
MP: membrane protein
MSP: membrane scaffold protein
PC: phosphatidylcholine
PE: phosphatidyl ethanolamine
PI: phosphatidylinositol
PS: phosphatidylserine
SM: sphingomyelin
TM: transmembrane.
